# Importing Diffusion and Re-Designed Backward Process for Image De-Raining

**DOI:** 10.3390/s24123715

**Published:** 2024-06-07

**Authors:** Jhe-Wei Lin, Cheng-Hsuan Lee, Tang-Wei Su, Che-Cheng Chang

**Affiliations:** Department of Information Engineering and Computer Science, Feng Chia University, Taichung City 407, Taiwan; lee2000061932@gmail.com (C.-H.L.); d0908831@o365.fcu.edu.tw (T.-W.S.); checchang@fcu.edu.tw (C.-C.C.)

**Keywords:** image process, diffusion model, de-rained, feature extraction

## Abstract

In recent years, with the increasing demand for high-quality images in various fields, more and more attention has been focused on noise removal techniques for image processing. The effective elimination of unwanted noise plays a crucial role in improving image quality. To meet this challenge, many noise removal methods have been proposed, among which the diffusion model has become one of the focuses of many researchers. In order to make the restored image closer to the real image and retain more features of the image, this paper proposes a DIR-SDE method with reference to the diffusion models of IR-SDE and IDM, which improve the feature retention of the image in the de-raining process, and then improve the realism of the image for the image de-raining task. In this study, IR-SDE was used as the base structure of the diffusion model, IR-SDE was improved, and DINO-ViT was combined to enhance the image features. During the diffusion process, the image features were extracted using DINO-ViT, and these features were fused with the original images to enhance the learning effect of the model. The model was also trained and validated with the Rain100H dataset. Compared with the IR-SDE method, it improved 0.003 in the SSIM, 0.003 in the LPIPS, and 1.23 in the FID. The experimental results show that the diffusion model proposed in this study can effectively improve the image restoration performance.

## 1. Introduction

Traditional rain removal methods are usually based on filtering and post-processing techniques. However, these methods are prone to the loss of detailed information and have limited effectiveness. In recent years, the rise of deep learning technology has injected new vitality into image rain removal. The diffusion model has attracted extensive attention from researchers as a generative model with a strong machine learning capability. It has shown superior performances in image generation and denoising. As a result, its application to rain removal tasks has emerged as a compelling and burgeoning research area.

In the diffusion model, the core idea is to gradually transform low-quality images into high-quality ones by simulating a random diffusion process. This approach is different from traditional models such as Generative Adversarial Networks (GANs), which focus more on simulating the physical process of image generation and are able to better deal with the details and textures in special scenes. Therefore, applying the diffusion model to the rain removal task is expected to achieve excellent results in improving the image quality of rainy-day photography. The diffusion model is a model with the ability to generate diverse high-resolution images and has been widely used in the field of image super-resolution in recent years. In 2015, Jascha Sohl-Dickstein and Eric et al. proposed the probabilistic diffusion model [1], when the authors completed the overall framework and mathematical derivatives. Demonstrating its excellent results in training and image reduction, Jonathan Ho et al. proposed denoising diffusion probabilistic models (DDPMs) in 2020 [2], which highlight the great potential of the diffusion model in the field of vision. Nowadays, this model has been gradually extended to various domains such as natural language processing (NLP), reinforcement learning (RL), and machine learning (ML). OpenAI’s DALL-E 2 [3] and Google’s Imagen [4] are both based on the diffusion model. Ziwei Luo et al. improved the traditional diffusion model by proposing a mean stochastic differential equation diffusion model that achieves better results in image denoising; the results are especially better in image denoising.

Despite the practical achievements of the diffusion model, there are still some challenges in the process of image generation, especially in the presentation of subtle features. These detailed deficiencies may affect the realism and quality of the generated images, so we wanted to make improvements to the diffusion model in order to enhance the overall quality of the generated images more effectively. We focused on solving the problem of possible detail deficiencies in the image generation process to improve the realism and quality of the images. The diffusion model has achieved remarkable success in generating images; however, its generated images are not satisfactory in terms of details. Therefore, the main objective of this study was to improve the detail reduction capability of the diffusion model-generated images in order to improve the visual quality of images captured in rainy weather or rainfall environments. Rainfall in rainy environments causes problems such as image blurring, contrast degradation, and color distortion, which is a challenge for many applications such as surveillance systems, autonomous vehicle technology, and remote sensing imagery. To solve this problem, the primary goal of this study was to develop an effective image de-rain enhancement method that focuses on preserving and enhancing subtle features in the image. These subtle features may include details, edges, textures, and structures, which are essential for image perception and understanding.

In order to accomplish this objective, In order to accomplish this objective, this study used diffusion models implemented in COMSOL Multiphysics (version 5.5), developed by COMSOL Inc., located in Burlington, MA, USA. These techniques have achieved excellent results in the field of image processing and some good results in the field of rain removal. By designing and training a diffusion model for rain removal, we hope to improve the restoration of images under the influence of rainfall. Careful consideration must be given to how to effectively label the subtle features of the image so that the model can better understand and restore these features without compromising the details and characteristics of the original image. Through this study, we anticipate offering an efficacious method for enhancing image quality, particularly in addressing unique scenarios such as rainy conditions.

## 2. Preliminaries

### 2.1. Image Restoration

In recent years, image restoration has been a major research area in computer vision, which is a broad and in-depth field aimed at improving visual quality by repairing, enhancing, or reconstructing damaged, blurred, or missing images. Research in this area has made significant progress in the past decades, involving a wide variety of techniques and approaches.

Initially, various CNN-based architectures were proposed for image super-resolution, denoising, and restoration. These methods can learn to extract features from large amounts of data and produce more realistic and clearer images. Among them, SRCNN [5] is one of the most classic models. SRCNN is used for single-image super-resolution and consists of three convolution layers that generate corresponding high-resolution images by learning the low-resolution representation of the image.

The subsequent VDSR [6] model further extends SRCNN by stacking deeper convolution layers to improve the performance. VDSR is mainly concerned with learning residual mapping, which makes it easier for the model to capture and preserve image details. DRCN [7], on the other hand, enables the model to perform image super-resolution at multiple stages by introducing a Recurrent Neural Network (RNN) structure. This recursive structure helps to better capture both local and global features in the image. EDSR [8] is a very deep super-resolution model that uses the structure of residual learning and a local residual block (LRCB) to improve the learning efficiency and model performance. EDSR has achieved excellent results in several super-resolution competitions. Generative adversarial networks have also been successfully applied to image super-resolution; SRGAN (Super-Resolution Generative) [9] introduces competition between discriminators and generators to produce more realistic and natural-looking high-resolution images.

This approach not only focuses on reconstruction details but also emphasizes that the generated image should resemble the real high-resolution image. In addition, there is a proliferation of task-specific models, such as DeblurGAN [10], which is a model applied to image deblurring that uses a GAN to learn clear image representations that can effectively deal with blurred images, such as problems caused by motion blur or anxiety blur.

### 2.2. Diffusion Model

A diffusion model is a model used to generate high-quality images that has attracted much interest in the field of image processing and restoration in recent years. The diffusion model is a probabilistic model that gradually changes the data into full noise by adding noise and learns to invert this process to generate new data.

The first proposed Diffusion Probabilistic Model (DPM) was proposed by Jascha Sohl-Dickstein and Eric et al. in 2015. The real application to computer vision and natural language was proposed by Jonathan Ho et al. in 2020 (Denoising Diffusion Probabilistic Models, DDPM). A DDPM is the first time that the generation of high-resolution images was debugged using two Markov chains; the first one is a forward process that adds noise to a picture, a reverse process that removes noise, and the second one is an inverse process that removes noise from a picture. The purpose of the forward process is to convert the data into a simple prior distribution, such as a Gaussian distribution, while the inverse process is used to reverse the forward process through deep learning and generate new data by sampling random vectors from the prior distribution. The DDPM can be further extended to infinite time steps where the denoising process is the solution of the stochastic differential equation (SDE). Therefore, we call this formulation the Score SDE [11] because it utilizes the SDE for noise disturbance and sample generation, and the denoising process requires the estimation of the score function of the noise data distribution.

In applying the diffusion process to generate high-resolution images with diversity, significant results have been achieved in training and image restoration. This research demonstrates the potential of the diffusion model in the visual domain and promotes the extension of the model’s application to various domains, such as natural language processing, reinforcement learning, and machine learning.

### 2.3. Score-Based SDE

The model simulates degradation from a high-quality image x(0) to a low-quality image μ+ϵ by diffusing the noisy version of the low-quality image. In simulating the corresponding inverse SDE, high-quality images can be recovered.

The forward process is defined as follows:

$$dx=\theta_{t}\left(\mu -x\right)dt+\sigma_{t}d\omega$$

Here, μ represents the state, which is the mean, where $\theta_{t}$ and $\sigma_{t}$ are time-dependent positive parameters representing the speed of the mean reversion and stochastic volatility, respectively. To perform image degradation, x(0) and μ are designated as the high-quality (HQ) and degraded low-quality (LQ) images, respectively.

To obtain a closed-form solution for the SDE, suppose that $\frac{\sigma_{t}^{2}}{\theta_{t}}=2\lambda^{2}$ holds for all time *t*. Then, given any state x(s) at time s<t, the solution of the SDE is

$$x\left(t\right)=\mu +\left(x\left(s\right)-\mu \right)e^{-{\bar{\theta }}_{s:t}}+\int_{s}^{t}\sigma_{z}e^{-{\bar{\theta }}_{z:t}}d\omega \left(z\right)$$

Here, ${\bar{\theta }}_{s:t}^{0}=\int_{s}^{t}\theta_{z}dz$ is known. The transition kernel $P\left(x|t\right)=N(x|m_{s:t}\left(x\left(s\right)\right),v_{s:t})$ is Gaussian, where the mean $m_{s:t}$ and variance $v_{s:t}$ are given by

$$m_{s:t}\left(x\left(s\right)\right)\mu +\left(x\left(s\right)-\mu \right)e^{-{\bar{\theta }}_{s:t}}$$

$$v_{s:t}\int_{s}^{t}\sigma_{z}^{2}e^{-2{\bar{\theta }}_{z:t}}dz=\lambda^{2}\left(1-e^{-2{\bar{\theta }}_{s:t}}\right)$$

When the initial state is x(0), we replace ${\bar{\theta }}_{0:t}$, $m_{0:t}$, and $v_{0:t}$ with ${\bar{\theta }}_{t}$, $m_{t}$, and $v_{t}$ respectively, and then we obtain the distribution of x(t) at any time *t* as

$$P_{t}\left(x\right)=N\left(x|m_{t}\left(\chi \right),v_{t}\right),$$

$$m_{t}\left(x\right)\mu +\left(x\left(0\right)-\mu \right)e^{-{\bar{\theta }}_{t}},$$

$$v_{t}\lambda^{2}\left(1-e^{-2{\bar{\theta }}_{t}}\right)$$

As t→∞, the mean $m_{t}$ converges to the low-quality image μ, and $v_{t}$ converges to $\lambda^{2}$. In other words, the forward SDE diffuses the high-quality image into a low-quality image with fixed Gaussian noise.

Backward Process

$$dx=\left[\theta_{t}\left(\mu -x\right)-\sigma_{t}^{2}\nabla_{x}\log p_{t}\left(x\right)\right]dt+\sigma_{t}d\hat{w}$$

The only unknown is the score function $\nabla_{x}\log p_{t}\left(x\right)$. Since during training we have access to high-quality images x(0), we can train a neural network to estimate the score $\nabla_{x}\log p_{t}\left(x\right)$. It can be computed as follows:

$$\nabla_{x}\log p_{t}\left(x|x\left(0\right)\right)=\frac{x\left(t\right)-m_{t}\left(x\right)}{v_{t}}$$

If we let $x\left(t\right)=m_{t}\left(x\right)+\sqrt{v_{t}}\epsilon_{t}$, where $\epsilon_{t}$ is Gaussian noise $\epsilon_{t}∼N\left(0,I\right)$,

$$\nabla_{x}\log p_{t}\left(x|x\left(0\right)\right)=-\frac{\epsilon_{t}}{\sqrt{v_{t}}}$$

Then, we follow the approach of using a noise network to approximate the noise, i.e., a conditionally dependent neural network ${\hat{\epsilon }}_{\phi }\left(\chi \left(t\right),\mu ,t\right)$ that takes state *x*, condition μ, and time *t* as input and outputs pure noise. Such a network can be trained using a loss similar to what is used in a DDPM:

$$L_{\gamma }\left(\phi \right)=\sum_{i=1}^{T}\gamma_{i}E[∥{\hat{\epsilon }}_{\phi }\left(x_{i},\mu ,i\right)-\epsilon_{i}∥]$$

where $\gamma_{1},\ldots ,\gamma_{T}$ are positive weights. After training, I can use the network ${\hat{\epsilon }}_{\phi }$ to sample noise state $x_{T}$ and iteratively solve using a numerical scheme.

### 2.4. Vision Transformer

The Transformer model was originally proposed by Vaswani et al. for machine translation and has become the most advanced method for many tasks in natural languages. Nowadays, it is applicable to many computer vision tasks, such as image recognition, image segmentation, and object detection. A Transformer decomposes an image into a series of patches and learns their interrelationships. The distinctive features of these models are their strong ability to learn remote dependencies between sequences of image blocks and their adaptability to a given input content. Due to these properties, Transformer models have also been investigated for low-level visual problems such as super-resolution, image coloring, noise removal, and rain removal.

SwinIR [12] is a special version of a Swin Transformer [13] dedicated to image restoration tasks such as super-resolution, noise removal, etc. SwinIR inherits the hierarchical structure of the Swin Transformer. A Transformer’s hierarchical structure helps to capture image details and global information at different levels. In addition, SwinIR introduces an attention mechanism to deal with long-distance dependencies in images. It is mainly applied to improve the quality of images, including super-resolution, denoising, and image restoration.

Uformer [14] is the Transformer model for de-raining. Uformer introduces two key components: a LeWin block and an MLP. The LeWin block is a variation of the Multihead Attention and MLP modules found in the Transformer architecture. It comprises two main elements: non-overlapping windows for self-attention and an MLP applied to tokens transformed into the feature map’s convolution layer.

## 3. The Improvement Procedure

The accuracy of the generated model is improved by replacing the general denoising stochastic diffusion model with a random differential equation and changing the forward noise addition process. Dino-ViT [15] utilizes a self-supervised model that has ViT as a teacher model to learn a lightweight student model and marks the attention map of the pictures, which facilitates the exchange of feature information. Therefore, based on these two foundations, this study proposes a hybrid framework of the two approaches to construct a new diffusion model.

**Figure 1:**
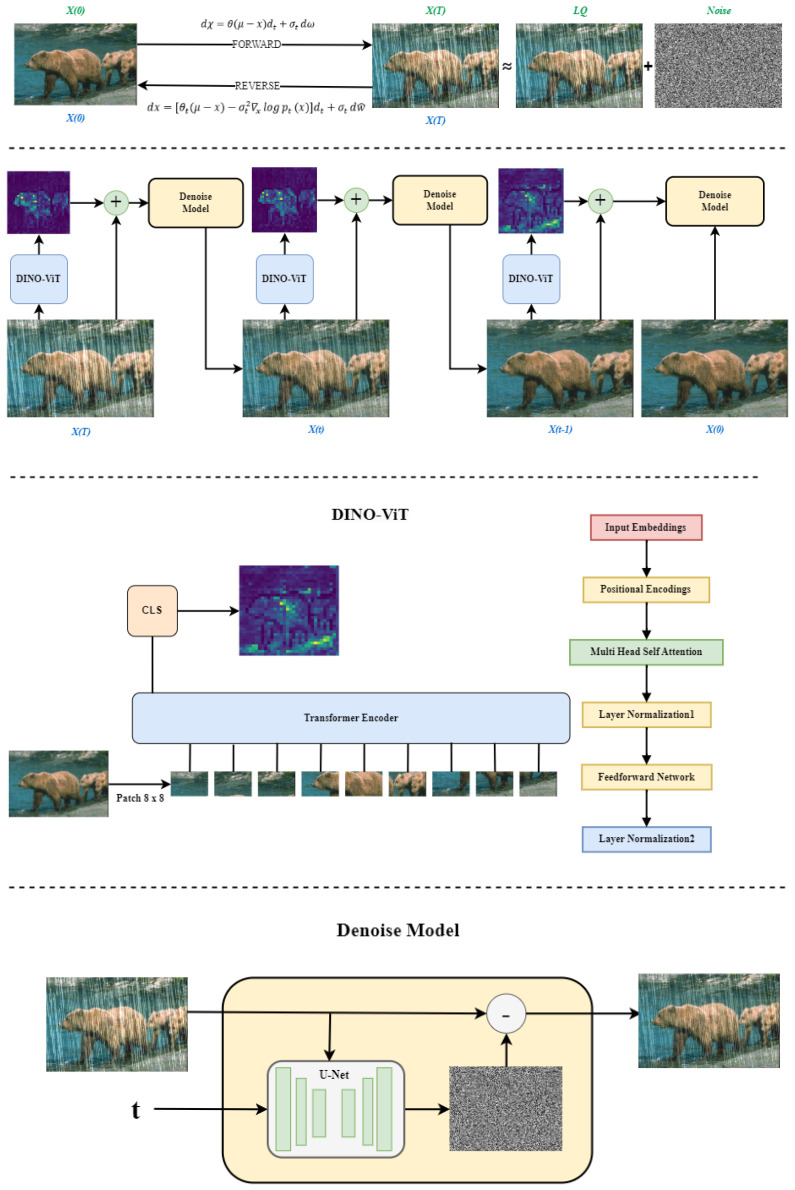
DIR-SDE model structure overview.

The model structure is shown in Figure 1. The model can be divided into three parts: diffusion models, image feature extraction, and image denoising. Diffusion models (diffusion models) aim to add noise to the image of the input image and gradually restore the image by learning to invert the process of adding noise. Image feature extraction aims to extract the features of the image so that the model can learn better; image denoising aims to restore the image with noise.

The DIR-SDE method is designed to denoise the image of the diffusion model in the inverse process to reduce the loss of features so that the image can be restored to the best results. As shown in Figure 1, the start of the diffusion model’s forward process will be with the real picture x(0) to add Gaussian noise to the picture x(T) where T is the total number of steps of the diffusion process at this time, and x(T) will be close to the low-quality picture LQ. Then, in the reverse process, the use of the forward process of adding noise to the process of learning the reverse process of gradual denoising to gradually restore our picture x(T) to picture x(0). This is the bi-directional process of the diffusion model, as shown in the upper part of Figure 1. In the reverse image derivation process, we extract the features of the image at each step to preserve the original features of the image and connect the obtained feature map with the original map. Then, denoising is performed to denoise the image and obtain the image x(t) at step x(t). t is the current step. Finally, the final generated image x(0) is obtained after T steps.

### 3.1. Diffusion Model

**Figure 2:**
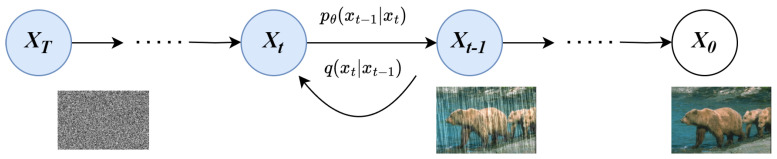
Diffusion process.

The core idea of diffusion modeling is to build a chance model that removes noise from an image over a series of time steps by means of a Markov chain. A Markov chain is a conditional probability distribution of future states of a stochastic process that depends only on the current state, given the current state and all past states. The diffusion model is divided into two-way Markov chains. The forward principle is to keep adding Gaussian noise to the picture so that the original picture gradually becomes completely noisy. The main method involves simulating a series of Gaussian noise distributions in the Markov chain to gradually add Gaussian noise to the original data, and then generate a signal that conforms to the Gaussian distribution, and then train a learning model that can gradually restore it. The algorithm consists of two steps, as shown in Figure 2. The process of gradually adding Gaussian noise to the data is called the diffusion process q(x). The process of gradually reducing the noise to the original data is called the reverse process p(x). Here, q(x) is not trainable, while p(x) is model-trained, and after training, we can obtain the learning model we want.

In the diffusion process using a fixed Markov chain, the current state is completely unrelated to the past state, and you can gradually add Gaussian noise to the data, given the initial data distribution q(x), and constantly add Gaussian noise to the distribution. The noise of the variance is $\beta_{t}$, and the average value of $\beta_{t}$ and the current t−1 moment of the data point $x_{t-1}$, $\beta_{t}$, are gradually increasing with the increasing noise, and we finally obtain $x_{T}$.

The process is written as follows:

$$q_{\theta }\left(x_{t}|x_{t-1}\right):=N\left(x_{t}:\sqrt{1-\beta_{t}}x_{t-1},\beta_{t}I\right)$$

The inverse diffusion process is performed to recover the initial data from the Gaussian noise process, which is still a Markov chain and can be assumed to be a Gaussian distribution, so the goal of the model is to learn the mean and the variance of the Gaussian distribution, and then to recover the data from $x_{T}$ to $x_{0}$. The process is written as follows:

$$p_{\theta }\left(x_{t-1}|x_{t}\right):=N\left(x_{t-1}:\mu_{\theta }\left(x_{t},t\right),\Sigma_{\theta }\left(x_{t},t\right)\right)$$

In this study, we utilized the stochastic differential equation (SDE) dispersion model as the main framework, which utilizes the Brownian motion, a continuous Gaussian process that adds a stable stochastic noise to the data at each successive stage. The basic stochastic differential equation process can be written as follows:

dx=f(x,t)dt+g(t)dω

where dω is the Gaussian noise, f controls the flow of diffusion throughout the stochastic differential equation, and g controls the degree of diffusion of the stochastic differential equation. In the stochastic differential equation, we call the above process a forward process, which corresponds to the process of adding noise, and we can change f to control whether the diffusion process finally becomes pure noise or data with noise, etc. The backward process is also the process of the stochastic differential equation. The inverse process is also the core of stochastic differential equations. Theoretically, any stochastic differential equation has a corresponding inverse, as shown in the following equation:

$$dx=\left[f\left(x,t\right)-g{\left(t\right)}^{2}\nabla_{x}\log p_{t}\left(x\right)\right]dt+g\left(t\right)d\hat{w}$$

where $\nabla_{x}\log p_{t}\left(x\right)$ is called the equation of the fraction of *x* at time *t*, and $\hat{w}$, which was originally additive noise, is now denoised. Here f and g are known, and only $\nabla_{x}\log p_{t}\left(x\right)$ is unknown, so we can evaluate this fraction in many ways and simulate the reverse process to recover the original data.

The mean-reverting stochastic differential equation (Mean-Reverting SDE) is called the mean-reverting stochastic differential equation (MRSDE) because it has one more μ than ordinary random differential equations, which means that the specified value or state is usually called the mean value. The following equation is used:

$$dx=\theta \left(\mu -x\right)dt+\sigma_{t}d\omega$$

The advantage of this is that after a certain number of steps, the whole stochastic differential equation flows to the mean value μ with stable Gaussian noise, which can be simply viewed as a gradual addition of noise to the data from x to μ. In the image recovery task, the image can be set to a low quality image. In the image recovery task, μ can be set to a low quality image. As long as there is an input and a real picture, the image degradation process can be established by means of mean-reverting stochastic differential equations, and the inverse process can be learned to recover the picture. The equation of the inverse process is as follows:

$$dx=\left[\theta_{t}\left(\mu -x\right)-\sigma_{t}^{2}\nabla_{x}\log p_{t}\left(x\right)\right]dt+\sigma_{t}d\hat{w}$$

**Figure 3:**
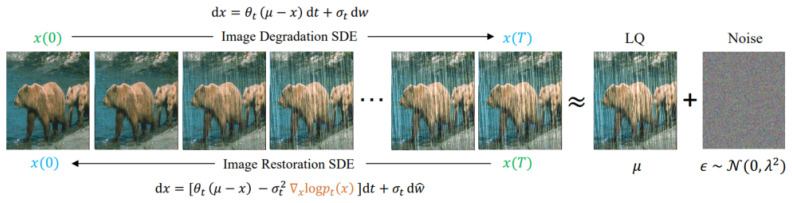
MSE process.

And the whole mean reversion stochastic differential equation is shown in Figure 3 as follows.

### 3.2. Reverse Denoising Process

**Figure 4:**
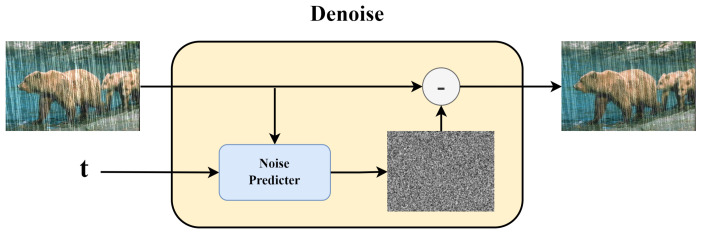
Noise predictor.

The inverse process in the diffusion model involves inputting a graph x(t) that we want to denoise and the corresponding noise predictor. This noise predictor is the model we want to train. The model will output the predicted noise, and finally, we subtract x(t) from the predicted noise to obtain x(t−1). This is shown in Figure 4.

**Figure 5:**
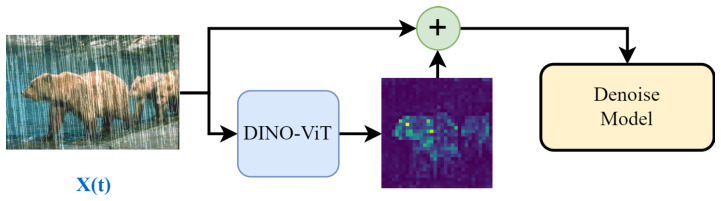
Denoising process.

Since, here, the original image is subtracted from the predicted noise, resulting in the original image possibly being damaged during the denoising process, this study wanted to find a way to ensure that the image would not be damaged during the denoising process. Inspired by the IDM [16], we first extract the features of the original graph and connect them with the original picture so that they will not be damaged too much in the denoising process, as shown in Figure 5.

### 3.3. DINO-ViT

In the field of computer vision, Transformers have recently become an alternative to CNNs. The Vision Transformer (ViT) has achieved results comparable to convolution networks but has not yet demonstrated sufficient advantages, as the ViT requires more computational resources and data. A major aspect of success comes from the application of self-supervised training. Self-supervised learning provides richer learning information than supervised learning. The richness of information in supervised learning images is reduced to concepts that correspond to a single subclass. Existing self-supervised learning has shown good results in images via convolutional networks. DINO-ViT was inspired by these findings to investigate the effects of self-supervised pretraining on ViT features, often by sharing similar structures while having different module designs to avoid overly trivial results or to improve effectiveness.

**Figure 6:**
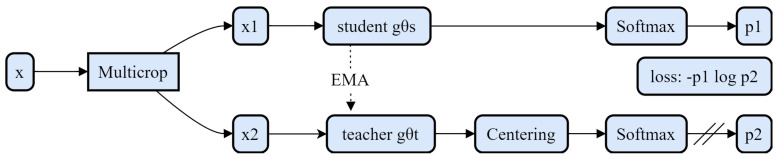
DINO-ViT knowledge distillation framework.

DINO-ViT utilizes a simple self-monitoring mechanism to design a label-free distillation method, as shown in Figure 6. The same graph is input, but two different stochastic transformations are performed to obtain $x_{1}$ and $x_{2}$, which are then given to the student network ($g_{\theta_{s}}$) and the teacher network ($g_{\theta_{t}}$). The two networks have the same structure but different parameters. The output of the teacher network is centered on the average of the batch calculation. Each network outputs a K-dimensional feature and is normalized using Softmax on the feature dimension. Their similarity is then measured using cross-entropy. A stop gradient is used for the teacher network, and only the gradient is propagated through the student network. The exponential moving average (EMA) of the parameters of the student network is used to update the parameters of the teacher network.

**Figure 7:**
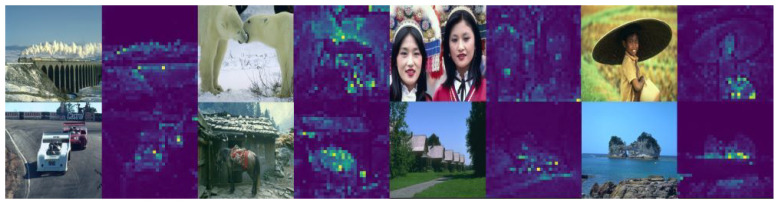
DINO-ViT attention map.

DINO-ViT can use an attention map to emphasize the key points. Labeling the key features in the patches allows the deep learning network to learn the areas to focus on in each picture, which also forms the attention map, as shown in Figure 7. Using the unsupervised method to train the self-attention of 8 × 8 patches for the ViT. We are looking at the self-attention of the last layer of a token. As shown in Figure 7, the model is able to learn class-specific features, thus realizing unsupervised class classification.

## 4. Experimental Results

In this experiment, we used the Peak Signal-to-Noise Ratio (PSNR), which is required for rain removal modeling; this metric is used to evaluate the quality of the images generated by the generative model, and its score calculates the mean square deviation of the pixel values of two photos. This study aimed to preserve the structure of images during the denoising process. To achieve this goal, we introduced Structural Similarity (SSIM), a metric used to compare images’ brightness, contrast, and structure. In addition, we adopted Learned Perceptual Image Patch Similarity (LPIPS), also known as perceptual loss, for evaluating the differences between two images. Compared with traditional methods (e.g., PSNR, SSIM), LPIPS is more in line with human perception and helps to capture subtle changes in image structure. In addition, we also introduced the Frechet Inception Distance score (FID), which is used to quantify the distance of feature vectors between the real image and the generated image. This provides a more comprehensive and sensitive evaluation of the differences between the generated image and the real image.

### 4.1. Data

**Figure 8:**
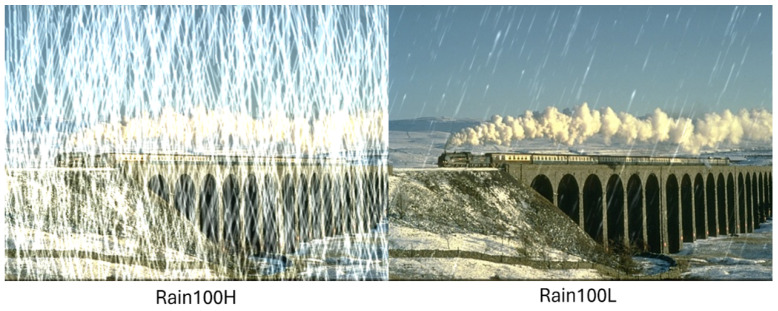
Rain100H and Rain100L.

The test dataset for the experiment is the synthetic rain dataset, which contains Rain100H and Rain100L, both of which contain 100 images, respectively. The difference between the two datasets is that Rain100H is a more complex dataset with more synthesized rain, while Rain100L is a less complex dataset with less synthesized rain, as shown in Figure 8.

### 4.2. Method

The other methods compared in this study have the same parameter settings as the best model settings in their individual papers. The comparison methods are listed below:

1. IR-SDE [17]: A method for using stochastic differential equations in extended dispersion models;
2. Restormer [18]: A method that uses the Transformer’s methods so that it can capture interactions between pixels;
3. MPRNet [19]: A method that uses the Multi-Stage Progressive method to produce spatially accurate and context-rich output;
4. PreNet [20]: A method involving a progressive optimized residual network progressive ResNet (PRN) and progressive optimized recurrent network progressive recurrent network (PReNet) for image de-raining;
5. M3SNet [21]: A method that improves of the U-net architecture to allow for more information exchange between up-sampling and down-sampling.

### 4.3. Results

As discussed in Section 4.3.1, a comparative analysis was conducted between the DIR-SDE method proposed in this study and other methodologies utilizing the Rain100H dataset. Subsequently, Section 4.3.2 presents the experimental outcomes of this method across various datasets, elucidating its performance characteristics.

#### 4.3.1. Comparison of DIR-SDE with Other Methods

Table 1 presents the comparative performances of various rain removal methodologies applied to the Rain100H dataset. An observation from Table 1 suggests that diffusion-based approaches, such as IR-SDE, exhibit superior efficacy compared to non-diffusion-based techniques like Restormer. This discrepancy underscores the beneficial impact of diffusion models in enhancing de-raining outcomes through bi-directional chaining and denoising mechanisms.

Our proposed method, DIR-SDE, demonstrates notable superiority across multiple evaluation metrics. Specifically, on the Rain100H dataset, our method achieves higher PSNR and SSIM scores compared to the Y channel baseline. Here, the Y channel represents the luminance component of the YCbCr color space, with Y denoting luminance and Cb and Cr denoting the blue and red chrominance components, respectively. Notably, compared to the runner-up method, DIR-SDE exhibits an improved SSIM score of 0.003 and an enhanced LPIPS score of 0.003, along with a reduction in FID by 1.23 while trailing in the PSNR by a mere 0.7. Despite a slight decrement in the PSNR, the overall performance of DIR-SDE remains commendable. It excels in preserving structural similarity and aligns closely with human visual perception.

In summary, while there may be room for improvement in the PSNR performance, the comprehensive efficacy of DIR-SDE across multiple evaluation criteria is noteworthy.

**Figure 9:**
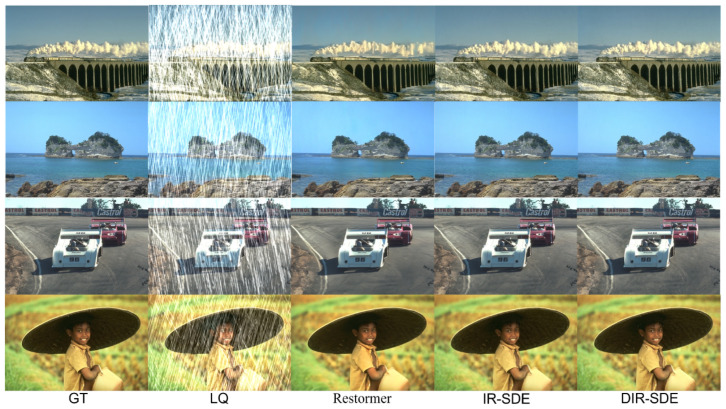
Visual results of our DIR-SDE with other rain removal methods at Rain100H.

Figure 9 presents a comparative analysis of visual outcomes achieved using different methodologies. Visual inspection reveals that DIR-SDE yields photographs with heightened realism and fidelity. Specifically, in Figure 9, it is evident that Restormer, which employs a Transformer for rain removal, fails to adequately preserve image features when compared to IR-SDE and our proposed method, both leveraging a diffusion model for rain removal. Notably, Restormer struggles to retain intricate details such as the smoke emitted by the train in the uppermost image of Figure 9, thereby hindering effective image restoration. Conversely, the diffusion modeling approach exhibits a superior preservation of the original image structure.

While IR-SDE demonstrates improved fidelity in overall image structure compared to previous methodologies, it still requires refinement in processing subtle image features. In contrast, our proposed method, DIR-SDE, excels in preserving subtle image features during the restoration process, without compromising on fidelity. This ensures that the denoising process does not lead to the loss of essential features.

**Figure 10:**
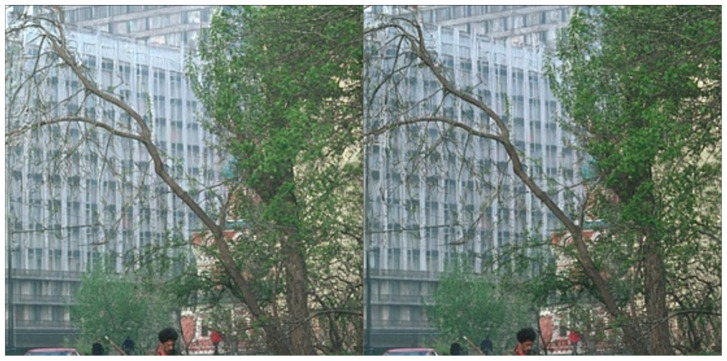
DIR-SDE is shown on the left. IR-SDE is shown on the rights.

Our DIR-SDE exhibits superior precision in the detailed processing of images compared to IR-SDE, as illustrated in Figure 10. Notably, our method demonstrates enhanced accuracy in preserving the structure of the pipeline outside the building, a task where IR-SDE blurs the pipeline due to the denoising process. This highlights our method’s ability to optimize the image denoising process while maintaining essential features without any loss.

**Figure 11:**
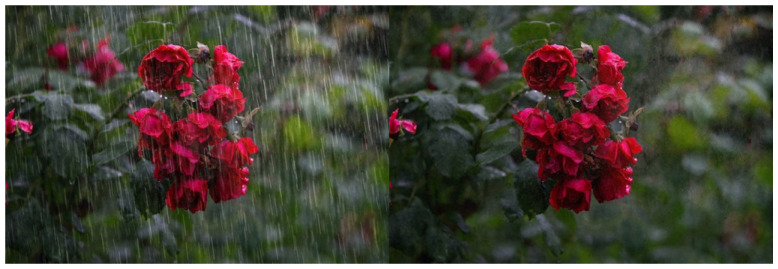
Applied to real world rain map I.

**Figure 12:**
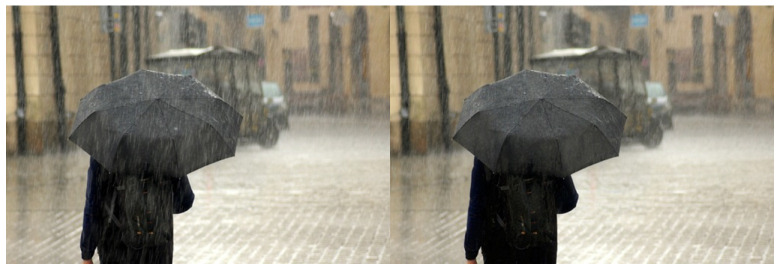
Applied to real world rain map II.

Furthermore, our method yields promising results when tested with real rain maps, as depicted in Figure 11 and Figure 12.

#### 4.3.2. Image Deblurring

To evaluate whether the method proposed in this paper can be applied to various image restoration tasks, we conducted experiments using the publicly available GoPro dataset [22], which contains 2103 training images and 1111 test images. The blurred images in the GoPro dataset are obtained through high-speed photography, providing pairs of real and blurred images. Compared to other blurred datasets, the GoPro dataset features more realistic and complex blurs.

**Figure 13:**
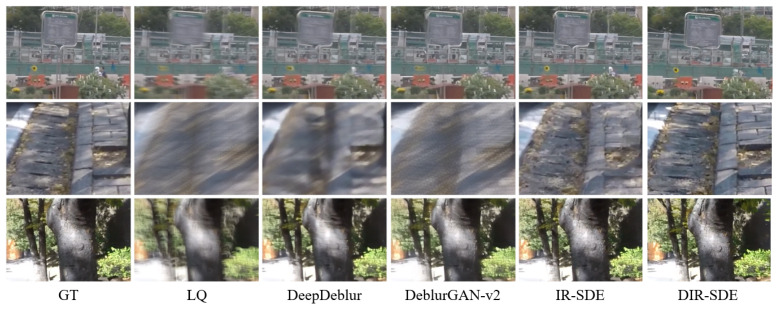
Visual results of our DIR-SDE with other deblurring methods on GoPro.

Table 2 presents the performance of each model on the GoPro dataset. For comparison, we selected four studies with outstanding results in the field of deblurring: DeepDeblur [22], DeblurGAN-v2 [23], DBGAN [24], and MAXIM [25]. In terms of the PSNR, the method proposed in this paper surpasses IR-SDE by 0.54 and DEBLURGAN-V2 by 1.69. This indicates that the images generated by DIR-SDE are closer to real images than those produced by other GAN-based methods. As illustrated in Figure 13, our method produces realistic results in the deblurring task.

**Figure 14:**
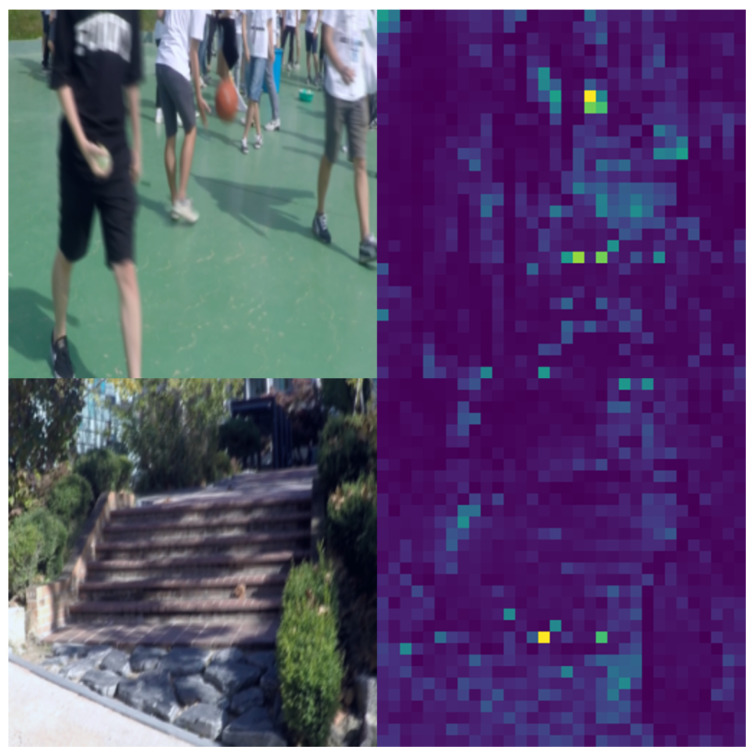
GoPro DINO-ViT attention map.

However, our method does not outperform IR-SDE in other metrics. This is primarily because, in the deblurring task when images undergo feature extraction by DINO-ViT, the blurriness and the fact that many images in the dataset depict scenes rather than distinct objects complicate feature extraction and structure recognition. As shown in Figure 14, DINO-ViT struggles to extract features effectively when characters are blurred and moving, though it performs better with scene images. This limitation hinders DINO-ViT’s ability to fully capture image features, thereby affecting the model’s performance in denoising tasks.

## 5. Conclusions and Future Work

In response to the outcomes of this experiment, the diverse metrics manifest a noteworthy level of performance. Although the PSNR value of the method proposed in this paper exhibits only a marginal deviation from the current state-of-the-art (SOTA) model, it is noteworthy that the PSNR value attained herein surpasses the threshold of 30, underscoring the method’s efficacy in image reconstruction. Furthermore, the method registers improvements of 6.3% and 6.6% in the LPIPS and FID metrics, respectively, corroborating its commendable performance in image restoration endeavors.

The impetus for this research emanates from the perpetual quest for advancing image restoration techniques, particularly the imperative to safeguard image features and structures. Inspired by IDM, we embarked on refining the IR-SDE architecture. Specifically, we integrated each step of the inversion process with an attention map generated by Dino-ViT. This novel configuration empowers our method to capture crucial image features and preserve structural integrity more effectively. Leveraging these attention maps enables our method to meticulously restore image details and structures, yielding images more faithful to reality.

Additionally, this study meticulously examined the processing efficacy of real raindrop images. Employing various datasets of authentic raindrop images, we subjected our method to rigorous testing. The results evince the method’s adeptness in handling authentic raindrop images, proficiently eliminating raindrops and reinstating clarity, substantiating the method’s feasibility and utility.

In conclusion, the DIR-SDE method delineated in this paper attained a commendable performance in the image restoration domain. Through its precise preservation of image features and structures and adeptness in processing authentic raindrop images, the method underscores its promise and significance in advancing the realm of image restoration.

## Figures and Tables

**Table 1:** Performance Comparison of Methods for Rain Removal Tasks.

Method	PSNR (↑)	SSIM (↑)	LPIPS (↓)	FID (↓)
DIR-SDE	30.95	0.9070	0.044	17.41
IR-SDE	31.65	0.9041	0.047	18.64
Restormer	31.46	0.904	-	-
MPRNet	30.41	0.8906	0.158	61.59
PReNet	29.46	0.8990	0.128	52.67
M3SNet	30.64	0.892	-	-

The ↑ indicates that a higher value is better, while ↓ indicates that a lower value is better.

**Table 2:** Performance Comparison of Methods for Image Deblurring Tasks.

Method	PSNR (↑)	SSIM (↑)	LPIPS (↓)	FID (↓)
DIR-SDE	31.24	0.8943	0.076	8.53
IR-SDE	30.70	0.9010	0.064	6.32
DEEPDEBLUR	29.08	0.9135	0.135	15.14
MAXIM	32.86	0.9403	0.089	11.57
DEBLURGAN-V2	29.55	0.9340	0.117	13.40
DBGAN	31.18	0.9164	0.112	12.65

The ↑ indicates that a higher value is better, while ↓ indicates that a lower value is better.

## Data Availability

Data will be available on reasonable request.

## References

[1] Sohl-Dickstein J., Weiss E.A., Maheswaranathan N., Ganguli S. Deep Unsupervised Learning using Nonequilibrium Thermodynamics. Proceedings of the 32nd International Conference on Machine Learning.

[2] Ho J., Jain A., Abbeel P. Denoising Diffusion Probabilistic Models. Proceedings of the Advances in Neural Information Processing Systems.

[3] Ramesh A., Dhariwal P., Nichol A., Chu C., Chen M. (2022). Hierarchical Text-Conditional Image Generation with CLIP Latents. arXiv.

[4] Saharia C., Chan W., Saxena S., Li L., Whang J., Denton E.L., Ghasemipour K., Lopes R.G., Ayan B.K., Salimans T. Photorealistic Text-to-Image Diffusion Models with Deep Language Understanding. Proceedings of the Advances in Neural Information Processing Systems 35 (NeurIPS 2022).

[5] Dong C., Loy C.C., He K., Tang X. (2016). Image Super-Resolution Using Deep Convolutional Networks. IEEE Trans. Pattern Anal. Mach. Intell..

[6] Kim J., Lee J.K., Lee K.M. Accurate Image Super-Resolution Using Very Deep Convolutional Networks. Proceedings of the 2016 IEEE Conference on Computer Vision and Pattern Recognition (CVPR).

[7] Kim J., Lee J.K., Lee K.M. Deeply-Recursive Convolutional Network for Image Super-Resolution. Proceedings of the 2016 IEEE Conference on Computer Vision and Pattern Recognition (CVPR).

[8] Lim B., Son S., Kim H., Nah S., Lee K.M. Enhanced Deep Residual Networks for Single Image Super-Resolution. Proceedings of the 2017 IEEE Conference on Computer Vision and Pattern Recognition Workshops (CVPRW).

[9] Ledig C., Theis L., Huszar F., Caballero J., Cunningham A., Acosta A., Aitken A., Tejani A., Totz J., Wang Z. Photo-Realistic Single Image Super-Resolution Using a Generative Adversarial Network. Proceedings of the 2017 IEEE Conference on Computer Vision and Pattern Recognition (CVPR).

[10] Kupyn O., Budzan V., Mykhailych M., Mishkin D., Matas J. DeblurGAN: Blind Motion Deblurring Using Conditional Adversarial Networks. Proceedings of the 2018 IEEE/CVF Conference on Computer Vision and Pattern Recognition (CVPR).

[11] Song Y., Sohl-Dickstein J., Kingma D.P., Kumar A., Ermon S., Poole B. (2021). Score-based generative modeling through stochastic differential equations. arXiv.

[12] Liang J., Cao J., Sun G., Zhang K., Gool L.V., Timofte R. SwinIR: Image Restoration Using Swin Transformer. Proceedings of the 2021 IEEE/CVF International Conference on Computer Vision Workshops (ICCVW).

[13] Liu Z., Lin Y., Cao Y., Hu H., Wei Y., Zhang Z., Lin S., Guo B. Swin Transformer: Hierarchical Vision Transformer using Shifted Windows. Proceedings of the 2021 IEEE/CVF International Conference on Computer Vision (ICCV).

[14] Wang Z., Cun X., Bao J., Zhou W., Liu J., Li H. Uformer: A General U-Shaped Transformer for Image Restoration. Proceedings of the 2022 IEEE/CVF Conference on Computer Vision and Pattern Recognition (CVPR).

[15] Caron M., Touvron H., Misra I., Jegou H., Mairal J., Bojanowski P., Joulin A. Emerging Properties in Self-Supervised Vision Transformers. Proceedings of the 2021 IEEE/CVF International Conference on Computer Vision (ICCV).

[16] Gao S., Liu X., Zeng B., Xu S., Li Y., Luo X., Liu J., Zhen X., Zhang B. Implicit Diffusion Models for Continuous Super-Resolution. Proceedings of the 2023 IEEE/CVF Conference on Computer Vision and Pattern Recognition (CVPR).

[17] Luo Z., Gustafsson F.K., Zhao Z., Sjölund J., Schön T.B. Image Restoration with Mean-Reverting Stochastic Differential Equations. Proceedings of the 40th International Conference on Machine Learning.

[18] Zamir S.W., Arora A., Khan S., Hayat M., Khan F.S., Yang M.-H. Restormer: Efficient Transformer for High-Resolution Image Restoration. Proceedings of the 2022 IEEE/CVF Conference on Computer Vision and Pattern Recognition (CVPR).

[19] Zamir S.W., Arora A., Khan S., Hayat M., Khan F.S., Yang M., Shao L. Multi-Stage Progressive Image Restoration. Proceedings of the 2021 IEEE/CVF Conference on Computer Vision and Pattern Recognition (CVPR).

[20] Ren D., Zuo W., Hu Q., Zhu P., Meng D. Progressive Image Deraining Networks: A Better and Simpler Baseline. Proceedings of the 2019 IEEE/CVF Conference on Computer Vision and Pattern Recognition (CVPR).

[21] Gao H., Yang J., Zhang Y., Wang N., Yang J., Dang D. (2023). A Mountain-Shaped Single-Stage Network for Accurate Image Restoration. arXiv.

[22] Nah S., Kim T.H., Lee K.M. Deep Multi-scale Convolutional Neural Network for Dynamic Scene Deblurring. Proceedings of the 2017 IEEE Conference on Computer Vision and Pattern Recognition (CVPR).

[23] Kupyn O., Martyniuk T., Wu J., Wang Z. DeblurGAN-v2: Deblurring (Orders-of-Magnitude) Faster and Better. Proceedings of the 2019 IEEE/CVF International Conference on Computer Vision (ICCV).

[24] Zhang K., Luo W., Zhong Y., Ma L., Stenger B., Liu W., Li H. Deblurring by Realistic Blurring. Proceedings of the 2020 IEEE/CVF Conference on Computer Vision and Pattern Recognition (CVPR).

[25] Tu Z., Talebi H., Zhang H., Yang F., Milanfar P., Bovik A., Li Y. MAXIM: Multi-Axis MLP for Image Processing. Proceedings of the 2022 IEEE/CVF Conference on Computer Vision and Pattern Recognition (CVPR).

